# Medical Items and News of Interest to the Physician

**Published:** 1870-01

**Authors:** 


### Medical Items & News.

### Of Interest to the Physician.

CULLED FROM THE STANDARD MEDICAL JOURNALS OF THE WORLD.

NOTE.—These formulæ are intended only for the regular practitioner of medicine, and should be employed only by him, or under his immediate supervision.

**Caffeine in Morphia Poisoning.**

Dr. Senneker, in the *St. Louis Med. Journal*, recommends the hypodermic injection of caffeine in poisoning from morphia. He mentions a case in which he injected a grain of pure caffeine, and after three grains had been injected, in ten minutes, the patient recovered.

**Syphilitic Warts.**

Use crystallized carbolic acid to syphilitic chancres and warts. Touch them with the acid every three days, and use lint saturated with a weak solution of the acid as a constant application. We have seen the worst form of chancre and wart disappear in one week, from this treatment.—*Ed. Bistoury.*

**Chlorate of Potash in Abortion.**

Dr. J. S. Lewis (*Med. and Surg. Reporter*) recommends the administration of chlorate of potash in threatened abortion.

He gives three grains four or five times a day. He cites several cases where this treatment prevented abortion when this misfortune was seriously threatened.

**Ether for Tape Worm.**

Dr. Lurtel (*Gaz. Med. de Paris*) uses with success the following method for expelling the tape worm: Two thirds of an ounce of ether is given in one dose, followed two hours afterwards by an ounce of castor oil. The worm is discharged entire, or almost so, and always with the head intact. No pain is caused by this treatment.

**Epilepsy.**

Prof. Da Costa's prescription for epilepsy, associated with irregularity of the heart, is as follows:

℞.

Zinci valerianatis, gr. iij.

Ext.belladonnæ, gr. ½.

Pulv. Digitalis, gr ¼.

For one pill. To be taken three times a day

**Diabetes.**

T. H. Tanner, M. D., F. L. S., of London, prescribes as follows:

℞.

Ferri ammoniæ citratis, ʒ j.

Spts. ammoniæ aromatici, f. ʒ vj.

Potassæ bicarbonatis, ʒ ij.

Infusi columbæ, q s. ad. f. ℥ iij.

M.

A teaspoonful to be taken twice a day, with one teaspoonful of lemon juice in a little water.

**Nervous Headache.**

℞.

Acidi nitro-muriatici diluti, f. ʒ ij.

Strychniæ, gr. ¼.

Spts. chloroformi, f. ʒ vj.

Tinct. Zingiberis, f. ʒ iij.

Aquæ, q. s. ad. f. ℥ iij.

M.

Sig:—A teaspoonful in water three times a day.

T. HAWKES TANNER, M. D., F. L. S., London.

**Constitutional Syphilis.**

Prof. William A. Hammond prescribes the following:

℞.

Potassii iodidi, ʒ j.
Hydrargyri chloridi corrosivi, gr. vj.
Aquæ, f. ℥ xij.
M.

Of this mixture a teaspoonful may be taken three times a day, till the system is well under its influence. Gradually increase the dose, so that after twenty or thirty days, a quarter of a grain of corrosive sublimate is taken three times a day.—*Ther. Bulletin.*

**Treatment of Scrofula.**

William Aitken, M. D., Edinburgh, prescribes as follows:

℞.

Olei morrhuæ, f. ℥ jss.

Olei creasoti, gtt. iv.

Pulv. tragacanthæ.

Pulv. accaciæ.

Pulv. amyli, āā ℈ j.

Sacchari albi, ʒ j.

Aqua anisi, f. ℥ ivss.

M.

Sig:—Take two tablespoonfuls three times a day. The creasote is said to render the stomach more tolerant of the remedy.—*Ther. Bulletin. Med. and Surg. Reporter.*

**Removal of Superior and Inferior Maxilliary Nerve for Facial Neuralgia.**

Dr. Mussey of Cincinnati removed the superior maxilliary nerve and the ganglion of Mechel, in a case of persistent facial neuralgia. In two months after, paroxysms of pain occurred in the lower maxilla, which became so severe that it was decided to remove the inferior maxilliary nerve also. This was done to the great relief of the patient.

---

**Infantile Syphilis.**

Prof. J. Lewis Smith, of New York, prescribes as follows:—

℞.
Hydrargyri cum creta, gr. iij.
Sacchari albi, ℈ j.
M.

Divide into twelve powders. One to be taken three times daily. It should not be discontinued entirely until several weeks after the syphilitic symptoms in the child have disappeared.—*Ther Bulletin, Med. and Surg. Reporter.*

---

**Spinal Irritation.**

Dr. Dutcher (*Cin. Med. Repository*) recommends in spinal irritation, the application of a succession of small blisters to the spine, and the following prescription:

℞.

Ext. Cannabis indica, ʒ ss.

Quiniæ valerian.,

Ext. scutillaria, āā ʒ j.

M.

and make thirty pills. Sig:—One pill every five minutes.

---

**Tilt's Suppositories.**

Edward John Tilt, M. D., London, (*Med. and Surg. Reporter*) uses the following suppository:

℞.
Ext. Hyoscyami, gr. jv.
" Belladonnæ, gr. $\frac{1}{3}$.

To be made round, coated with cocoa butter, and introduced at night. It relieves pain without constipating.

---

**Ointment for Hæmorrhoids.**

Prof. Fordyce Baker, of Bellevue Hospital Med. College, recommends the following ointment in hæmorrhoids:

℞.

Unguentum Gallæ Comp., ℥ j.

Plumbi Acetas, ʒ j.

Morph. Sulphas, ℈ j.

M.

**Functional Indigestion.**

The following is prescribed by Wm. Aitken, M. D., Edin.:

℞.
Sodæ bicarbonatis, gr. xv.
Potassæ nitratis, gr. iij.
M.

For one powder, to be taken two or three times a day, in those forms of indigestion marked by excessive acidity and heartburn.

Another:

℞.
Ferri redacti,
Pepsinæ, āā gr. xxxvj.
Zinci phosphatis, gr. xviij.
Glycerinæ, q. s.

Divide into twenty-four pills. Two to be taken every day at dinner. In anæmia, etc., with weakness of the digestive organs.

---

**Carbolic Acid in Caries.**

The *N. Y. Medical Journal* has the following,—A patient, domestic, aged 78, applied with extensive caries of the tibia, the leg having numerous sinuses between the knee and ankle, with copious discharges therefrom, through each of which bone could be felt with a probe. The supposed cause was tertiary syphilis. Eight grains of iodide of potassium, three times daily were ordered; and the sinuses in the leg were freely syringed out with a solution of carbolic acid ʒ ss. to the pint of water. In six weeks the leg was perfectly well, and her general health had so much improved as to enable her to go about her domestic service as usual.

---

**How to Cauterize the Cervix Uteri.**

In cases of ulceration of the cervix uteri or in any difficulty of the parts where cauterization is necessary, the following method has succeeded admirably in our hands:—Take an ordinary sponge tent and dip it into hot bees-wax; when cool roll it, by means of a spatula, in powdered nitras argenti, until the surface of the bees-wax is completely covered with the powder. Then, through your speculum introduce your tent into the cervix, and if desirable, to the fundus, and allow it to remain twelve hours.

In chronic inflammation, engorgement, enlargement, or ulceration of the *os* and *cervix uteri*, this is the very best application that we have ever tried.—*Ed. Bistoury.*

---

**Hemlock in Chorea.**

Chorea or St. Vitus's Dance, is often relieved by the administration of the juice of conium.

**Radical Cure of Varicocele.**

Mr. Bartwell is in the habit of separating the vas deferens and spermatic arteries from the veins, keeping the former back and the latter forward. He then passes a needle, armed with annealed iron wire, behind the veins—between them and the vas deferens—beginning on the outer side of the scrotum, and bringing it out on the inner side of the cord. Then the needle is re-entered at the orifice of exit, passed in front of the veins, and brought out at the point where it first entered. A loop of wire is thus formed, passing completely around the veins, and *inside* the scrotum, but involving no part either of skin or fascia.

The proceeding is almost painless and perfectly safe. The wire is twisted gradually every day until the vessels are severed and the loop brought away in the course of a week. The scrotum must be suspended in a bandage, and the patient kept quiet.

---

**Strumous Ophthalmia of Children.**

We are in the constant habit of prescribing as follows:

R.
Olei Morrhuæ, f. ℥ viij.
Tinct. ferri chloridi, f. ℥ ij.
M.

Shake thoroughly and give a child of five years, half a teaspoonful of the mixture, three times daily, before eating. This should be continued for three or four months, and the eyes washed with the following collyrium, morning and evening, until all ulceration has subsided:

R.

Acidi carbolici. (Sol.), gtt. v.

Glycerinæ, f. ʒ ss.

Aqua Rosæ, f. ℥ j.

M.

We have treated hundreds of cases at the Elmira Eye and Ear Institute, and have always succeeded with the above formulæ, varying them only to suit special cases.—*Ed. Bistoury.*

---

**Irrigation in treatment of Wounds, &c.**

Mr Isaac Walton, (*Lancet*), believes *irrigation* superior to carbolic acid or any other form of dressing, when there is sloughing or danger of pyæmia. Instead of the foul materials which are generated being allowed to accumulate, as they do in a poultice, and undergo decomposition, they are washed away as soon as they are formed.

His apparatus consists of a small reservoir, from which hang some worsted threads; the water is thereby conveyed to the diseased part, over which is laid a peice of linen, with the lower edge hanging free, so that there can be no impediment to the escape of the fluids and the foul materials. A gutta-percha sheet receives the drippings and conveys it to a vessel on the floor. He has never used carbolic acid or any drug with the water.

The editor of the *Med. Archives*, treated a case in August, of compound fracture of the arm, and compound comminuted fracture of the fore-arm, with a very satisfactory result, which was attributed mainly to *irrigation* with water slightly impregnated with liq. sod. chlorinat., sustained by an apparatus similar to the one above described. But for the antiseptic effect of the irrigation, amputation, he thinks, would have been unavoidable.

---

**Puerperal Fever.**

A physician writing to the *Med. Archives*, has had great success in treating puerperal fever in the following manner:—First fomentations over regions of uterus, continued twelve hours, renewed every thirty minutes; simple mush in winter, or peach tree leaf in season. Veratrum (Norwood's) dose, seven to ten drops; intervals two hours, and alternate with spiritus terebinth. ℥ j., dulcis nitr. ℥ ij. M., of this ʒ ss to the dose. At same time rinse the uterus with uterine syringe, with terpid water and sweet cow's milk, every four or five hours. In from ten to twelve hours a remission of fever with free perspiration ensues. Then omit the veratrum, and substitute balsam copavia, fifteen to twenty drops, every two hours, and inject the uterus as follows:—quinine, forty grains; tepid water, six ounces. Give a full dose of paregoric. Let the patient rest five hours, then give five grains of quinine with morphia or opium. In the last fifteen years he says he has not seen a case die under this mode of treatment. During the exhibition of veratrum and for several hours, the child must not nurse from the mother's breast.

---

**De-Nicotinized Tobacco.**

A distinguished physician writing to the *Chicago Med. Journal*, thus discourses upon Tobacco: It is not generally known that tannic acid de-nicotinizes tobacco. If the bowl of the pipe is filled about one-fourth full of tannin, filled up with tobacco and smoked, the aroma of the tobacco is almost entirely destroyed, and the smoker scarcely feels the effect of the tobacco in his nervous system.

We all know what inveterate smokers Indians are, and still we never see any injurious effects of this habit upon them. This is due to the form in which they smoke their tobacco. They rarely or never use the pure leaf. Their "Killikinnick" is composed of equal parts of tobacco and the inside bark of a species of the *cornus coricea*. Sometimes the admixture of tobacco is not more than a fourth. This bark is an astringent, and abounds in *tannin*, and therefore in a great measure neutralizes the effects of the tobacco. The fancy brands of smoking tobacco labelled "Killikinnick," sold by tobacconists in papers, are pure tobacco and have no real claim to the name. The Indian name for the particular species of swamp dogwood which they use for smoking, is "Kinnickinnick"—hence the name. The Indians peel the inside bark of the shrub, dry it, pound it to a powder in their stone mortars, and then mix intimately with the crumbled tobacco.

---

**Nocturnal Enuresis and Incontinence of Urine.**

Dr. Snelling of New York, in the *Med. Gazette*, says that this affection is much more common in girls than boys, and that it is desirable to acertain the absence of all vaginal discharge, worms, or other sources of rectal or vaginal irritation. When the disorder has arisen from over-distention of the bladder, there may result an affection of the nerves, or of the muscular fibre, or both, inducing paralaysis of the viscus, and allowing it to fill up without the knowledge of the patient, and to dribble away drop by drop. This condition is more likely to occur in after life and from injury, than in early youth. An excellent prescription for this class of cases is the following:

℞

Elixir Aromat. Secalis,

Tr. Uva Ursi, āā ℥ ss.

Tr. Buchu, ℥ vj.

Ess. Pyrolæ, ʒ ij.

Syrup Zingiberis, ℥ j.

M.

Sig:—Half or one teaspoonful three times daily, for an adult.

In those cases dependent on atony or paralaysis of the sphincter vesicæ, the sheet anchor of treatment is as in the following formula:

℞.

Ext. nucis vomicæ, gr. viij.

Oxydi ferri nigri, ʒ j.

Pulv. quassiæ, ʒ j.

Syrup. absinth., q. s.

M.

Fiat massa et pillula xlviij. dividendum.

Sig:—One pill to be taken three times daily.

**Rheumatism.**

The following article is taken from the *Med. and Surg. Reporter*, and is the treatment adopted by Thomas King Chambers, M. D., London: Our author calls rehumatic fever "a pleasant disease for the doctor to treat, though not for the patient to bear," and gives a very simple, uniform plan of treatment, which, he states, hardly ever requires modification. *Bedding*:—The patient's bed is made in a peculiar fashion. No linen should touch the skin. A slight calico shift or shirt may be allowed; but if the patients possess underclothing only of the prohibited sort, they are better naked. Even a linen front to the shirt is dangerous. The sheets should be removed and the body carefully wrapped in blankets, the newest and fluffiest that can be got. The head is to be carefully protected from currents of air. *Fomentations:*—Those joints or limbs which are swollen, red, or painful are to be wrapped up in flannels, soaked either in hot water or in a decoction of poppy heads, with half an ounce of carbonate of soda to each pint.

*Curative Drugs:*—If the skin is red, swollen, and painful about the joints, if motion is impossible or the cause of exquisite suffering, and especially if these phenomena are metastatic, then the "alkaline treatment" is employed, as follows:—

℞.

Potassæ bicarbonatis, ℈ j.

Aqua camphoræ, f. ʒ ij.

M.

For one dose, to be repeated every three hours, day or night, when awake. If, however, the above symptoms are insignificant, and the pain is felt more in the bones, being intensified by pressure rather than by motion, and fixed not metastatic, then two grains of the iodide of potassium are to be added to each dose. So soon as the symptoms take a favourable turn, the alkali is to be omitted altogether and only the iodide of potassium given.

*Palliatives*:—Opium is to be administered in amounts proportionate to the subjective sensation of pain—from one to two grains at a dose. Immediately upon the relief of the pain, the quantity is diminished. If the pain remain fixed in one joint after it has left other places, leeches are to be applied there and the part kept poulticed. Bruised laural leaves may be mixed with the poultice. If the heart become affected, leeches and poultices are to be applied to the cardiac region.

*Diet:*—The food is to be varied to some extent by the social and personal state of the patients. If they have been hearty and well-to-do persons before the attack, simple diet is proper, *i. e.*, bread and butter, gruel and tea. if they have been ill-nourished, a pint of broth or of beef-tea is added. Meat even during convalescence often does harm, seeming to turn into lactic acid. Vegetable food should be pretty closely adhered to in order to avoid a relapse.

### Peas and Beans.

These articles have been found by chemical analaysis rich in nitrogen. The inference has been that they would be specially useful in supporting the waste of the muscles of animals and it has been suggested that they would be particularly useful in the production of wool. They are evidently valuable for these purposes, but not less valuable for the production of fat. Those persons who have used peas for fattening hogs, consider them worth as much as Indian corn. In districts where that grain is not grown, very fine pork is produced from peas. Dickson, in his work "On the Breeding of Live Stock," states that a sweep stake was entered into between five East-Lothian farmers, to be claimed by the one who should be pronounced the best feeder of cattle. Forty cattle of the same breed, and in equal condition, were divided between them, as fairly as possible. They were put up together the second week of September, and killed at Christmas following. The winner of the stakes fed his animals wholly on boiled beans with hay.

### Deafness.

A correspondent in Victor county, Texas, says he received a circular some time since from a Dr. Stillwell, of New York, in which he proposes to cure deafness, or refund the money. Our correspondent wishes to know if the aforesaid Stillwell is a first-class physician, and can do what he proposes to.

He may be a first-class physician, able to make the ears of a tin pail sound, for all we know, but he has never been heard of in New York, so far as we can learn. If he can do as he professes, it will not be necessary for him to send out circulars, for he can have more work in this city alone than will give him constant employment, and enrich him for life.

First-class physicians are not in the habit of spending their time addressing circulars to people all over the country.—*Pomeroy's Democrat.*

A good lecture on Quackery, "Brick."

